# Hydrolysis of Extracellular Pyrophosphate increases in post-hemodialysis plasma

**DOI:** 10.1038/s41598-018-29432-4

**Published:** 2018-07-23

**Authors:** Daniel Azpiazu, Emilio González-Parra, Jesús Egido, Ricardo Villa-Bellosta

**Affiliations:** Fundación Instituto de Investigación Sanitaria, Fundación Jiménez Díaz (FIIS-FJD), Avenida Reyes Católicos 2, 28040 Madrid, Spain

## Abstract

Vascular calcification (VC) is associated with significant morbidity and mortality of dialysis patients. Previous studies showed an association between loss of plasma pyrophosphate and VC. Moreover, loss of pyrophosphate occurs during dialysis in this population, suggesting that therapeutic approaches that prevent reduction of plasma pyrophosphate levels during dialysis could improve the quality of life of dialysis patients. This study found that pyrophosphate hydrolysis was 51% higher in post- than pre-dialysis plasma. Dialysis sessions modified the kinetic behavior of alkaline phosphatase, increasing its *V*_*max*_ and reducing its *K*_*m*_, probably due to the elimination of uremic toxins during dialysis. At least 75% of alkaline phosphatase activity in human plasma was found to depend on a levamisole-sensitive enzyme probably corresponding to tissue non-specific alkaline phosphatase (TNAP). Dialysis increased total plasma protein concentration by 14% and reduced TNAP enzyme by 20%, resulting in an underestimation of pyrophosphate hydrolysis in post-dialysis plasma. Levamisole inhibited TNAP activity (IC_50_, 7.2 µmol/L), reducing pyrophosphate hydrolysis in plasma and increasing plasma pyrophosphate availability. Alkaline phosphatase is also found in many tissues and cells types; therefore, our results in plasma may be indicative of changes in phosphatase activity in other locations that collectively could contribute significantly to pyrophosphate hydrolysis *in vivo*. In conclusion, these findings demonstrate that dialysis increases pyrophosphate hydrolysis, which, taken together with previously reported increases in alkalization and calcium ion levels in post-dialysis plasma, causes VC and could be prevented by adding calcification inhibitors during dialysis.

## Introduction

Vascular calcification is a common complication in hemodialysis patients and is associated with cardiovascular events and all-cause mortality^1^. Hyperphosphatemia is a typical clinical manifestation in these patients. Increases in plasma phosphate levels have been associated with the prevalence of calcification^2^ due to the spontaneous formation of calcium-phosphate crystals^3^. Moreover, calcification has been found to contribute to the substantial morbidity and mortality rates in this patient population^4,5^.

There are two major consequences regarding the fate of vascular smooth muscle cells in phosphate-induced calcification^6,7^. The first involves a profound transition to a bone-forming phenotype, that results in the loss of vascular smooth muscle cells markers and the expression of osteochondrogenic markers^8,9^. The second consequence invokes apoptosis-dependent matrix mineralization, which has been detected both in cultured humans vascular smooth muscle cells^10,11^ and in arteries from pediatric dialysis patients^12^.

Pyrophosphate is the main endogenous inhibitor of calcium-phosphate crystal formation and growth *in vitro*^13–15^ and *in vivo*^16–19^. Reductions in plasma pyrophosphate concentrations have been associated with vascular calcification^20^. Pyrophosphate is generated enzymatically via the hydrolysis of extracellular ATP by the enzyme ectonucleotide pyrophosphatase/phosphodiesterase (eNPP)^21^, and pyrophosphate is degraded to inorganic phosphate (Pi) mainly by tissue non-specific alkaline phosphatase (TNAP)^22^. Overexpression of TNAP in vascular smooth muscle cells is sufficient to cause *ex vivo* calcification in aortic rings^22^, and murine models with increased expression and activity of TNAP have demonstrated excessive vascular calcification^22,23^.

Reductions in plasma pyrophosphate levels after dialysis^24,25^ may be due to increases in phosphatase activity^25^. To expand on these findings, this study analyzed the kinetic behavior of alkaline phosphatase activity in plasma from hemodialysis patients, the effect of dialysis on pyrophosphate hydrolysis, and the effect of alkaline phosphatase inhibition on pyrophosphate availability.

## Results

### Alkaline phosphatase kinetic behavior in plasma is altered by dialysis

**Figure 1:**
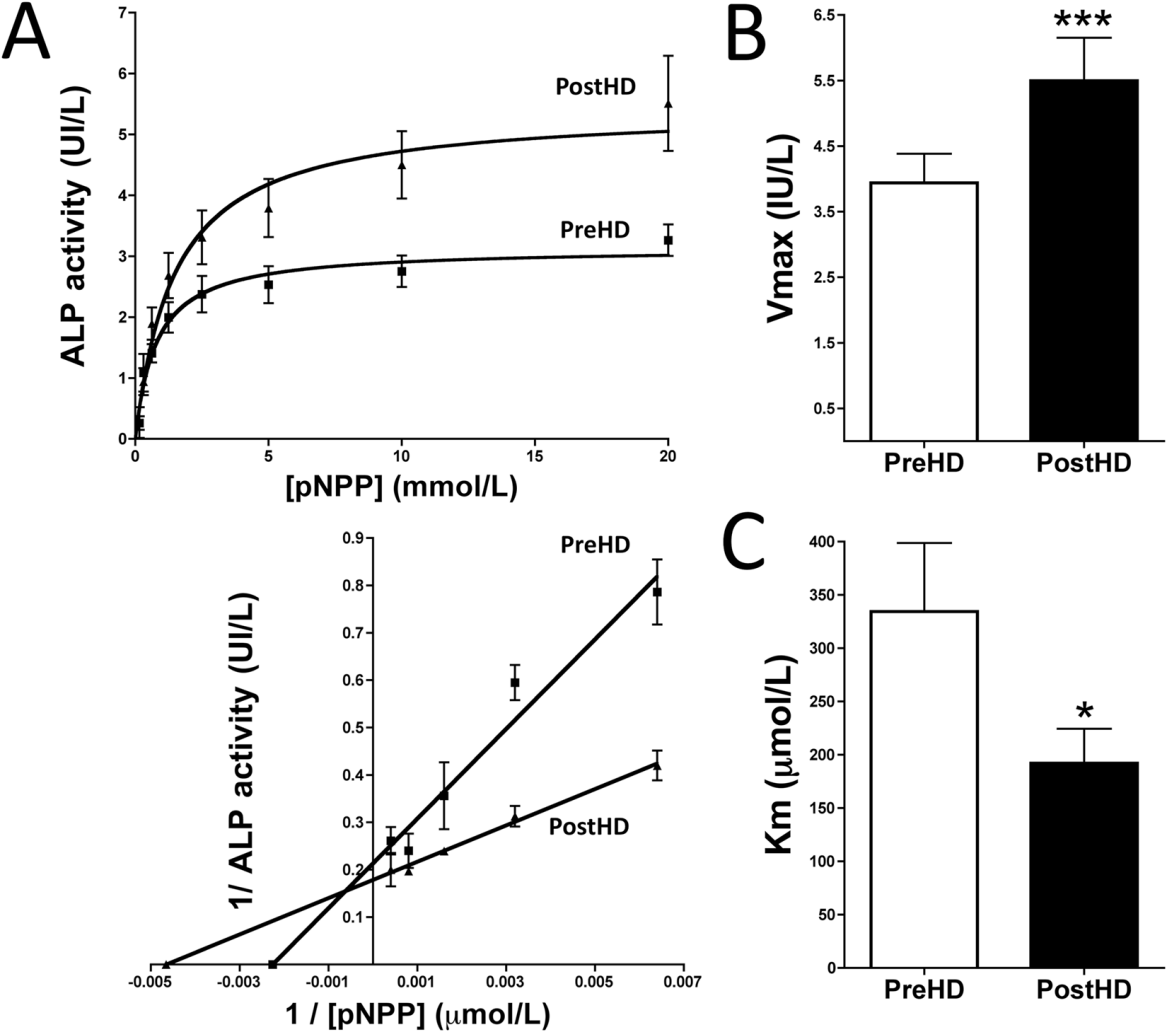
Kinetic analysis of ALP activity in plasma samples before and after dialysis. (**A**) Representative Michaelis-Menten saturation curves to determine the *K*_*m*_ and *V*_*max*_ of pNPP hydrolysis in pre- and post-dialysis plasma samples under physiological conditions (top). Representative Lineweaver-Burk plot (bottom). The curves for each patient were used to determine *V*_*max*_ (**B**) and *K*_*m*_ (**C**) using nonlinear regression, as described in the Methods section. Results are presented as mean ± SEM (n = 40), and were compared by the Wilcoxon matched pairs test. *P < 0.05; ***P < 0.001.

To analyze the kinetic behavior of plasma alkaline phosphatase, saturation kinetics for p-nitrophenyl phosphate (pNPP) hydrolysis in 40 pairs of samples were fitted to a Michaelis-Menten equation, V = (V_max_ S)/(K_m_ + S), where V is the velocity of pNPP hydrolysis, V_max_ is the maximal velocity or capacity of pNPP hydrolysis, S is the concentration of pNPP and K_m_ is the affinity constant. Analysis of the enzyme kinetics of plasma alkaline phosphatase showed that its *V*_*max*_ was ∼40% higher (5.50 ± 0.66 IU/L vs. 3.94 ± 0.44 IU/L, *P* < 0.001) and its apparent *K*_*m*_ was significantly lower (192.0 ± 32.5 μmol/L vs. 334.5 ± 64.2 μmol/L, *P* < 0.05) after than before dialysis (Fig. 1).

### TNAP is the main phosphatase in human plasma

**Figure 2:**
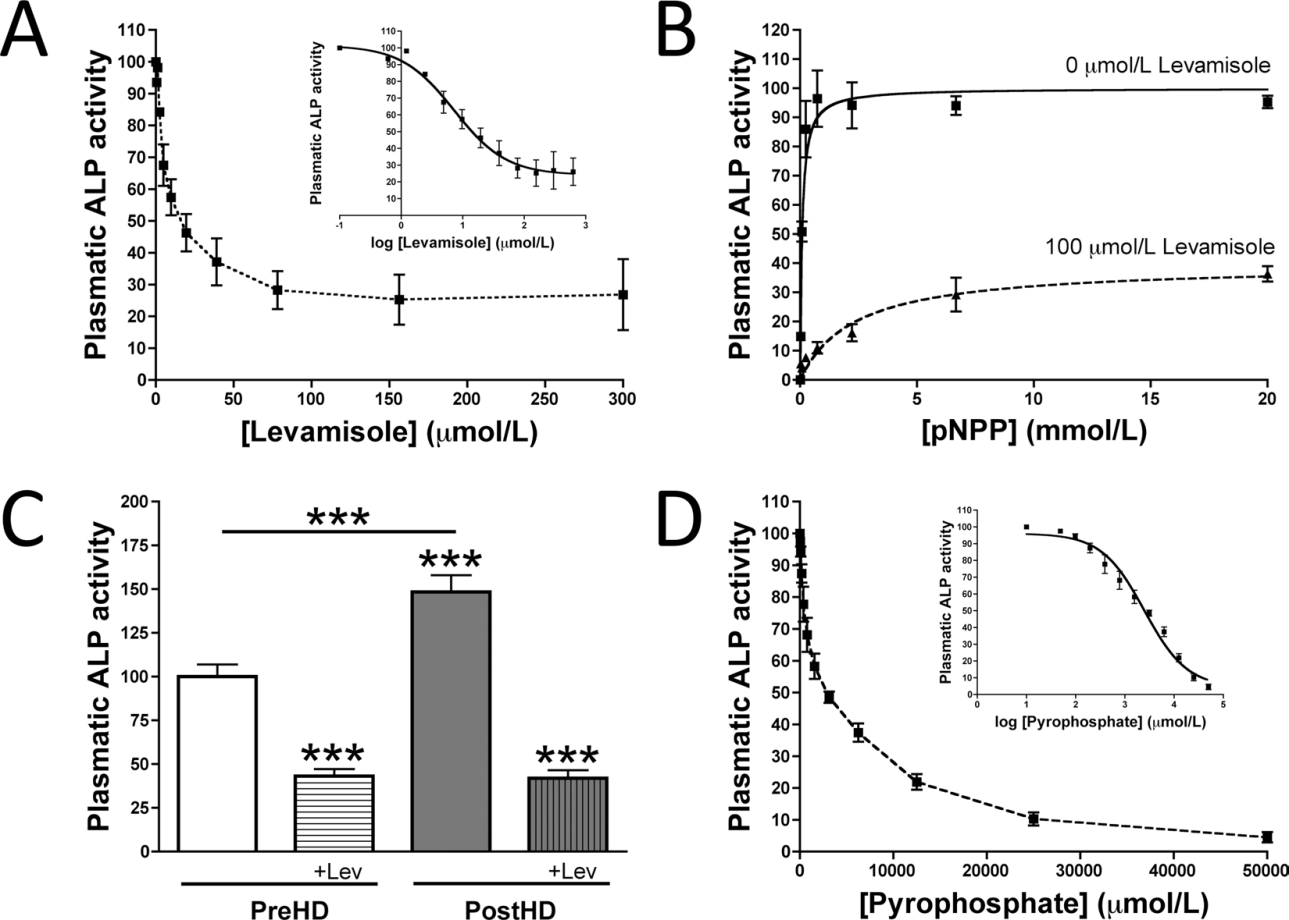
Tissue non-specific alkaline phosphatase (TNAP) is the main phosphatase in human plasma. (**A**) Kinetic characterization of levamisole inhibition of pNPP hydrolysis. (**B**) Michaelis-Menten saturations curves to determine the *K*_*m*_ and *V*_*max*_ of plasma pNPP hydrolysis in the absence (−) or presence (+) of levamisole. (**C**) Plasmatic ALP activity in pre- and post-hemodialysis plasma (PreHD and PostHD, respectively) in absence and presence of 100 µmol/L levamisole (+Lev). (D) Kinetic characterization of pyrophosphate inhibition of pNPP hydrolysis. Results are presented as mean ± SEM of nine pools of post-hemodialysis plasma samples in three independent experiments.

To ascertain the effect of alkaline phosphatase inhibitors on the availability of plasma pyrophosphate, we first analyzed the inhibitory kinetics of levamisole, a known inhibitor of TNAP activity, in human plasma. In this case pNPP has been also used as substrate. Levamisole had an IC_50_ of 7.2 µmol/L, with a concentration of 100 µmol/L completely inhibiting alkaline phosphatase activity (Fig. 2A). A dose-response analysis of plasma ALP substrate after hemodialysis showed that 100 µmol/L levamisole reduced the *V*_*max*_ of ALP activity to 24% of that of the control (Fig. 2B,C) shown that the levamisole-sensitive phosphatase is the main component increased after dialysis. Finaly, pyrophosphate had an IC_50_ of 2477 µmol/L, which correspond with a *K*_*i*_ (*K*_*m*_ for pyrophosphate) of 611.9 µmol/L pyrophosphate (Fig. 2D).

### Plasma pyrophosphate hydrolysis increases following dialysis

**Figure 3:**
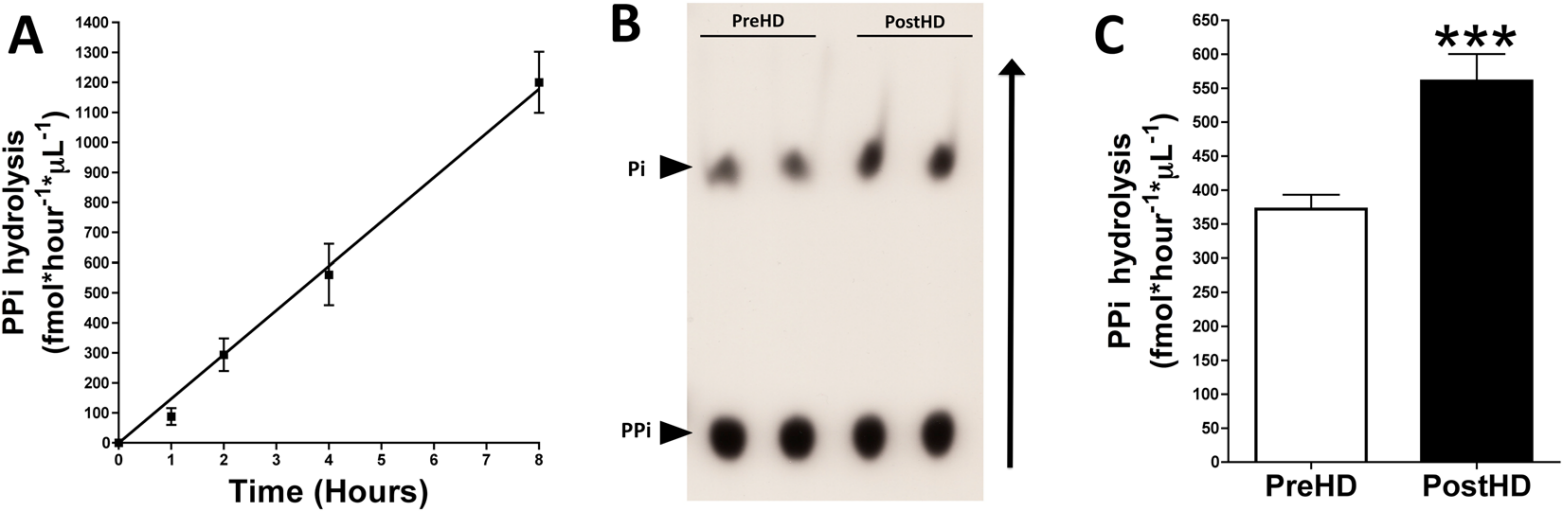
Pyrophosphate hydrolysis in plasma is higher in patients after than before dialysis. Plasma samples obtained before and after dialysis were incubated for for the indicated time with 5 μmol/L pyrophospahte and 32-pyrophosphate (^32^PPi) as a radiotracer. A 5 μL aliquot of each sample was separated by thin layer chromatography. After radiography, the spots were excised and added to liquid scintillation fluid. Pyrophosphate hydrolysis was quantified as percent 32-phosphate (^32^Pi) produced relative to total CPM (^32^Pi + ^32^PPi) and represented as fmol pyrophosphate hydrolyzed per hour and per μL of plasma sample (fmol*hour^−1^*μL^−1^). (**A**) Pyrophosphate hydrolysis in post-hemodialysis plasma for the indicated time. Results are presented as mean ± SEM (n = 9). (**B**) Representative radiography of a thin layer chromatograph showing ^32^Pi and ^32^PPi after incubation for 4 hours. (**C**) Pyrophosphate hydrolysis in plasma samples before (PreHD) and after (PostHD) dialysis after incubation for 4 hours. Results are presented as mean ± SEM (n = 40), and were compared by the Wilcoxon matched pairs test. ***P < 0.001.

Pyrophosphate hydrolysis was quantified as 32-phosphate (^32^Pi) released from the hydrolysis of 32-pyrophosphate (^32^PPi) in plasma. ^32^Pi and ^32^PPi were separated by chromatography on PEI-cellulose plates and counted by liquid scintillation. ^32^PPi hydrolysis in plasma was linear over 8 hours (Fig. 3). After 4 hours of incubation, ^32^PPi hydrolysis in plasma was 51% higher after than before dialysis (11.2% ± 5.0% vs. 7.4% ± 2.7%, *P* < 0.001; Fig. 3). This correspond with a plasma pyrophosphate hydrolysis of 370 fmol * hour^−1^ * µL^−1^ (pre-dialysis) and 560 fmol * hour^−1^ * µL^−1^ (post-dialysis).

### Inhibition of TNAP activity increases pyrophosphate availability

**Figure 4:**
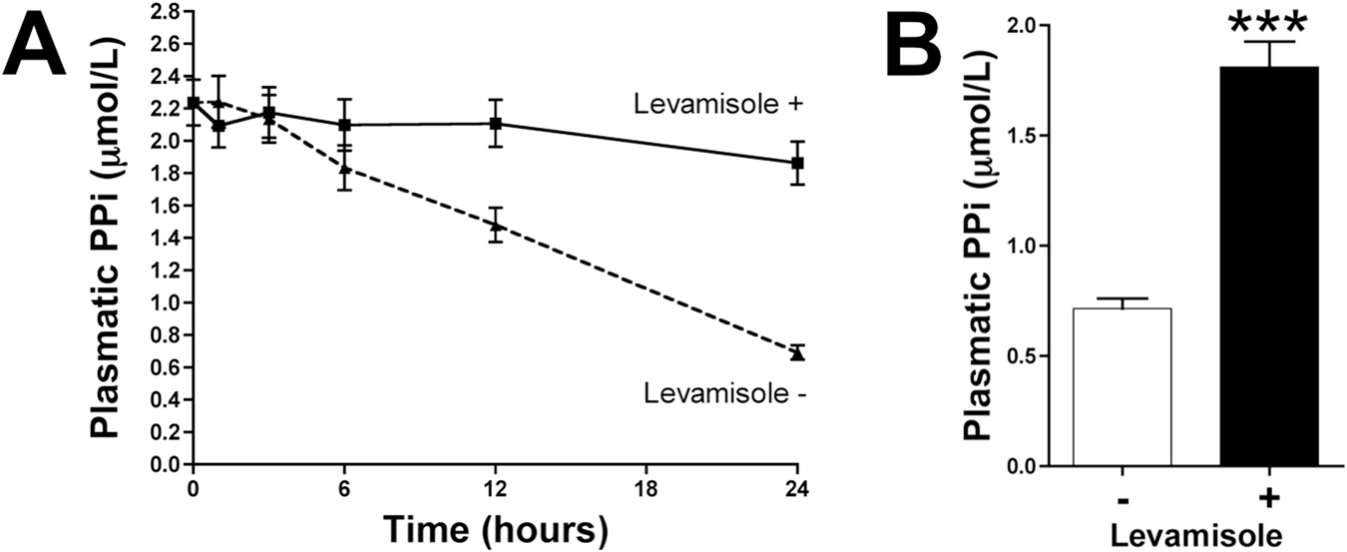
Inhibition of tissue non-specific alkaline phosphatase increases pyrophosphate availability in human plasma. Post-hemodialysis plasma samples were incubated at 37 °C in the absence (−) or presence (**+**) of 100 µmol/L levamisole. (**A**) Quantification of plasma pyrophosphate levels (n = 8) at the indicated times. (**B**) Quantification of pyrophosphate levels in post-hemodialysis plasma samples (n = 40) after incubation for 24 hours. Results are presented as mean ± SEM, and were compared by the Wilcoxon matched pairs test. ***P < 0.001.

Addition of 100 µmol/L levamisole to post-hemodialysis plasma samples reduced pyrophosphate hydrolysis over time (Fig. 4A) and increased pyrophosphate availability after 24 hours (Fig. 4B). In the absence of levamisole, plasma pyrophosphate was markedly hydrolyzed.

### Loss of TNAP despite an increase in protein concentration after dialysis

Plasma protein concentration was found to be ~14% higher after than before dialysis (7.34 ± 0.22 g/dL vs. 6.42 ± 0.19 g/dL, *P* < 0.001; Fig. 4A). Coomassie Brilliant Blue staining revealed similar findings after proteins were separated by molecular weight (Fig. 4B). Immunoblotting with TNAP antibody showed that the plasma concentration of this ∼53 kDa protein was significantly lower after than before dialysis (∼13%; *P* = 0.0063; Fig. 4C and Supplementary Info File). Moreover, TNAP quantification by ELISA (Fig. 4D) showed that the level of this protein was ~20% lower after than before dialysis (27.59 ± 1.10 µg/dL vs. 34.43 ± 0.62 µg/dL, *P* < 0.001; Fig. 4D).

### Phosphate inhibit pyrophosphate hydrolysis in rat aortic wall

Aortas were obtained from euthanized rats and digested over 10 min in order to remove the adventitia layer. Then, pyrophosphate hydrolysis assay in absence of phosphate was first performed. Then, after washing five times in MEM media without phosphate, the same aortic rings were used for pyrophosphate hydrolysis assay in presence of 1 mmol/L phosphate. Pyrophosphate hydrolysis was found to be 3.7-fold higher (Fig. 6) in absence of phosphate (5.50 ± 0.18 pmol * mg^−1^ * min^−1^) that in presence of phosphate (1.53 ± 0.17 pmol * mg^−1^ * min^−1^).

## Discussion

Reductions in plasma pyrophosphate levels, which occur following hemodialysis^24,25^, have been associated with vascular calcification^16,20^. Because vascular calcification is the main clinical adverse effect in dialysis patients, largely determining their morbidity and mortality rates, further exploration of these findings may improve patient quality of life. This study showed that the reduction in plasma pyrophosphate levels following dialysis could be probably due to an increase in pyrophosphate hydrolysis. The ~51% increase in pyrophosphate hydrolysis was due primarily to increases in plasma alkaline phosphatase activity following dialysis. We found that the *V*_*max*_ of this enzyme increased by ∼40%, while its *K*_*m*_ decreased by ∼40%, from before to after dialysis. These findings are compatible with the presence of both competitive and non-competitive inhibitors, which are removed from plasma during dialysis. For example, the elimination of phosphate from plasma during dialysis^25^ may explain, at least in part, the increase in levamisol-sensitive alkaline phosphatase activity. Moreover, since alkaline phosphatase is found in many tissues and cells types (anchored in the cell membrane), pyrophosphate hydrolysis in isolated plasma is much less than *in vivo*. However, our results in plasma may be indicative of changes in phosphatase activity in other locations that collectively could contribute significantly to pyrophosphate hydrolysis *in vivo*. This could also explain the associated up-regulation of TNAP enzyme in uremic aorta shown in previously studies^16^, as a compensatory mechanism to improve the loss of hydrolysis capacity due to the phosphatase inhibition with uremic toxins (mainly phosphate).

Interesting, high levels of plasma alkaline phosphatase are also associated with mortality in all stages of chronic kidney diseases. Our study revealed an increase in alkaline phosphatase activity in post-dialysis plasma. Therefore, this hidden consequence of hemodialysis increases our knowledge of the factors contributing to mortality in this population.

We also found that levamisole, an inhibitor of TNAP^22^, had an IC_50_ value of 7.2 µmol/L, with a concentration of 100 µmol/L completely inhibiting alkaline phosphatase activity in human plasma. Interestingly, 75% of alkaline phosphatase activity in plasma is provided by a levamisole-sensitive enzyme, probably TNAP^22^. Although levamisole has been used as an anthelmintic treatment agent in humans, it has been replaced by more effective treatments. Levamisole could be used to prevent excessive pyrophosphate hydrolysis during dialysis sessions while developing more effective TNAP inhibitors. We found that the addition of levamisole to post-dialysis plasma reduced the hydrolysis of pyrophosphate, thereby increasing its availability.

Although we found that the increased pyrophosphate hydrolysis in post-dialysis plasma was associated with an increase in phosphatase activity, the plasma concentration of TNAP was ∼20% lower after than before dialysis. These findings suggest that ~51% higher pyrophosphate hydrolysis in plasma after than before dialysis is an underestimate. During dialysis, low molecular weight proteins are lost (<60 kDa). These may include TNAP, with a molecular weight of ∼53 kDa, suggesting that the lower TNAP concentration after than before dialysis session may be the consequence of diffusion during dialysis.

In conclusion, this study showed that pyrophosphate hydrolysis in plasma is greater after than before dialysis, despite the reduction in the level of TNAP protein, the main phosphatase in human plasma. Moreover, reduction in pyrophosphate levels is also be influenced by increments in tissue/cell TNAP activity. Because reductions in plasma pyrophosphate levels are associated with vascular calcification^20^, the findings of this study indicate that a loss of ability to prevent calcification plays a predominant role during this pathological process^16,26^. Vascular calcification is therefore associated with reductions in plasma pyrophosphate levels^24,25^ and increases in alkalization^25,27^ and calcium concentrations following dialysis. Supplementation with exogenous anticalcifying agents, such as pyrophosphate and TNAP inhibitors, may therefore inhibit or prevent dialysis-associated calcification.

## Material and Methods

### Hemodialysis conditions and sampling

Each patient underwent a conventional, purely diffusive 4 hour (mid-week) hemodialysis session without hemodiafiltration, using a high flux helixone dialyzer (Fresenius; CUF, 59 mL/h/mmHg; surface, 1.8 m^2^). The dialysate was composed of 1.5 mmol/L calcium, 35 mmol/L bicarbonate, 0.75 mmol/L potassium, 0.5 mmol/L magnesium, and 140 mmol/L sodium.

**Figure 5:**
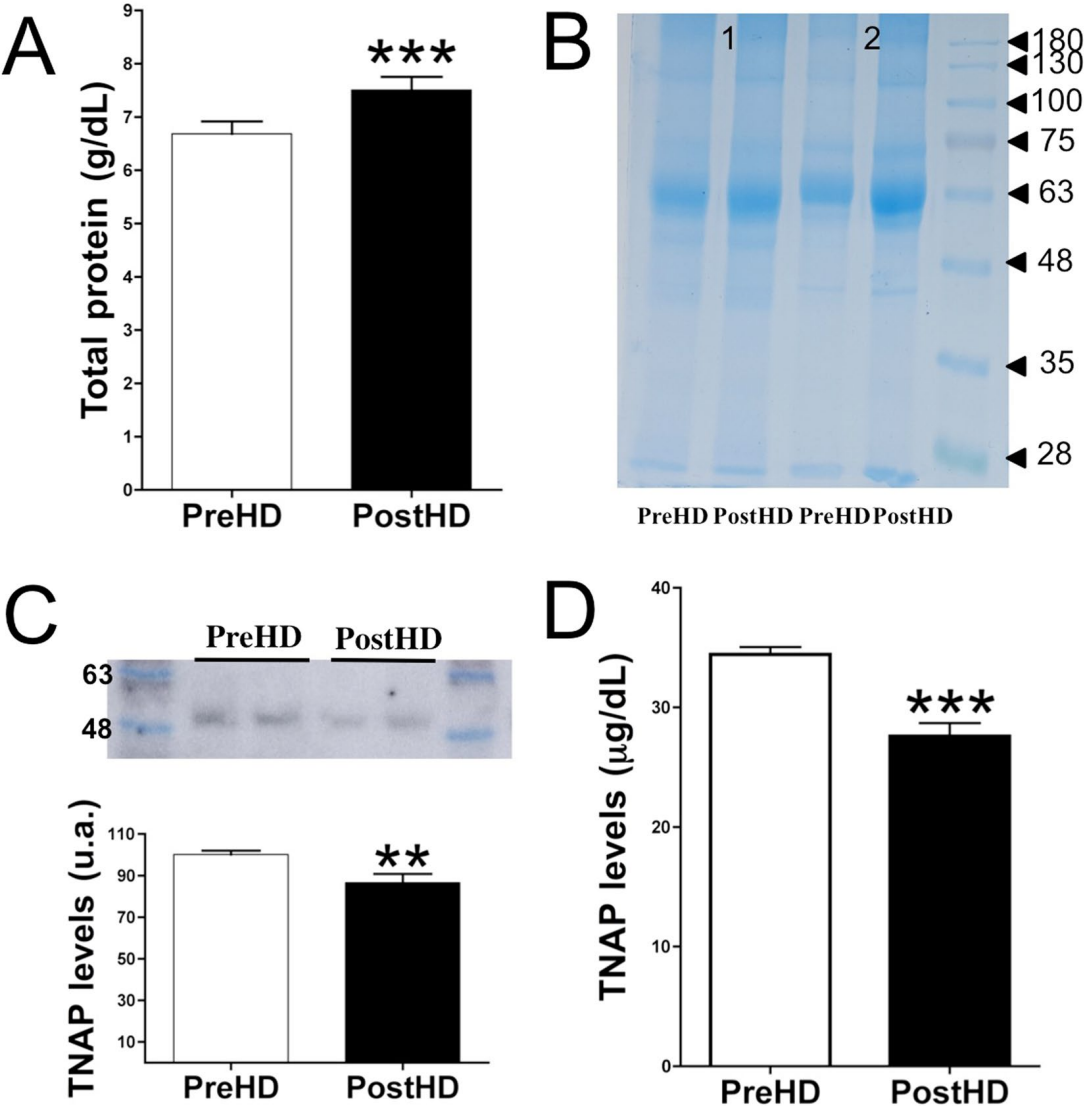
Loss of TNAP protein in post-dialysis plasma. (**A**) Total protein concentrations in plasma samples before and after dialysis. (**B**) Representative Coomassie Brilliant Blue staining of plasma proteins in two pairs of samples. (**C**) Representative immunoblot of TNAP levels in plasma samples before and after dialysis (upper), and relative level of TNAP level in post-dialysis relative to pre-dialysis samples (lower). (**D**) ELISA quantification of TNAP levels in plasma samples before and after dialysis. Results are presented as mean ± SEM of 40 pairs of samples, and were compared using the Wilcoxon matched pairs test. **P < 0.01; ***P < 0.001.

Blood samples before and after hemodialysis were collected in heparin-containing tubes and centrifuged at 5000 rpm for 5 min at 4 °C. In the case of Fig. 5 (pyrophosphate quantification) heparin-containing tubes were immediately centrifuged at 5000 rpm for 5 min at 4 °C. Plasma samples were frozen in liquid nitrogen and stored at −80 °C until further use. This study was conducted according to the Declaration of Helsinki and was approved by the Ethics Committee of Research of University Hospital Fundación Jiménez Díaz. Participants, ranging in age from 46 to 80 years and consisting of 26% women, were identified by a number and no other identifying material. Subjects were included if they were adults on stable chronic hemodialysis with a life expectancy over 6 months according to clinical criteria and provided informed consent. There were no exclusion criteria based on compliance; calcium, phosphate, parathyroid hormone, or vitamin D levels; or concomitant medications. Patients with positive serology for HIV, HB surface Ag, or HCV or other known active infection were excluded.

### Pyrophosphate hydrolysis and quantification

Plasma samples (5 µL) were incubated in 15 µL Molecular Biology Water (BE51200, Lonza, Switzerland) containing pyrophosphate (S6422, Sigma-Aldrich, St. Louis, MO) and [^32^P]pyrophosphate (Perkin Elmer, Boston, MA) at final concentrations of 5 µmol/L and 10 µCi/mL, respectively. After 4 hours, the samples were chromatographed on PEI-cellulose plates (105579; Merck, Germany), which were developed with 650 mmol/L K_2_HPO_4_ (P5655, Sigma-Aldrich) pH 3, as described^22^. After radiography, the spots containing phosphate and pyrophosphate were removed and added to liquid scintillation fluid (UltimaGold^™^, 6013329; Perkin Elmer). Radioactivity was measured using the liquid scintillation analyzer Tri-Carb 2810TR (Perkin Elmer).

Pyrophosphate was measured using an enzyme-linked bioluminescence assay, as described^16,25^. After measuring pyrophosphate levels in post-dialysis plasma samples, these samples were incubated in the presence or absence of levamisole for up to 24 hours, and pyrophosphate was again quantified.

### Alkaline phosphatase activity and inhibition

Alkaline phosphatase activity was measured using pNPP (N4645, Sigma-Aldrich) as substrate. Briefly, 50 µL of human plasma was incubated with 150 µL of dH_2_O containing 0–20 mmol/L pNPP for 2 hours, and the absorbance of the solution was measured at 405 nm every 30 min. To test the ability of levamisole (31742, Sigma-Aldrich) or pyrophosphate to inhibit TNAP activity, 50 µL of human plasma was incubated with 150 µL of dH_2_O containing 10 mmol/L pNPP and the indicated levamisole or pyrophosphate concentration. Slopes and activities were calculated by linear regression using GraphPad Prism 5 software.

### Protein quantification, gels, and immunoblots

Plasma protein levels were quantified using a BCA Protein Assay Kit (Pierce, Rockford, IL). Briefly, plasma samples were prepared in loading Buffer (62.5 mmol/L NaCl, 1 M Tris, pH 6.8; 2% SDS; 10% glycerol; 0.05% bromophenol blue), and the same volume for each pair of samples (containing 20 to 50 μg of protein) was separated by SDS-PAGE and stained with Coomassie Brilliant Blue (Generon; Maidenhead, UK) or blotted to polyvinylidenedifluoride (PVDF) transfer membranes (Immobilon^®^-P; Merck Millipore Ltd, Billerica, MA) as described^16^. The membranes were incubated with primary rabbit polyclonal anti-TNAP (1 μg/mL, ab65834, Abcam) antibody. After incubation with the appropriate secondary antibody (GENA934, Sigma-Aldrich), the blots were developed using Luminata™Classic Western HRP Substrate (WBLUC0500, Millipore, Billerica, Massachusetts) in the ImageQuant LAS 4000.

TNAP levels in plasma were quantified using ELISA kits for alkaline phosphatase in liver, bone, and kidney (SEB091Hu, Wuhan USCN Business Co., Ltd.; Houston, TX).

### Kinetic analyses

Alkaline phosphatase saturation kinetics for pNPP hydrolysis were fitted to a Michaelis-Menten equation, V = V_max_ S/(K_m_ + S), where V is the velocity of pNPP hydrolysis, V_max_ is the maximal velocity or capacity of pNPP hydrolysis, S is the concentration of pNPP, and K_m_ is the affinity constant.

The mean inhibitory concentration (IC_50_) was also calculated by nonlinear regression using the one-site competition equation, V = Bottom + [(Top-Bottom)/(1 + 10^(S-logIC50)^)], where Top refers to the velocity of pNPP hydrolysis in the absence of inhibitor (levamisole), and Bottom refers to the maximal inhibition.

The inhibition constant, K_i_, for pyrophosphate (its K_m_) was then calculated indirectly using the following equation: K_i_ = IC_50_ /[1 + (S/K_m_)], were S is the constant concentration of pNPP (0.250 mmol/L) and K_m_ is the affinity constant of pNPP (0.082 mmol/L).

### Animals

Male Sprague-Dawley rats (8–12 weeks) were obtained from Charles River Laboratories (France). The protocol was approved by ethics committees both the FIIS-FJD (Fundación Instituto de Investigación Sanitaria, Fundación Jiménez Díaz) and Madrid Community (PROEX 427/15); and conformed to directive 2010/63EU and recommendation 2007/526/EC regarding the protection of animals used for experimental and other scientific purposes, enforced in Spanish law under RD1201/2005.

### Aorta isolation and pyrophosphate hydrosysis assay

Rats were euthanized via carbon dioxide inhalation and thoracic aorta tissue was perfused with saline and removed according to previously published protocols^15^Then, the pyrophosphate hydrolysis assay was performed.

**Figure 6:**
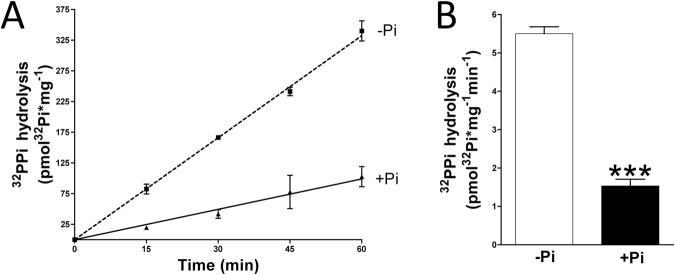
Phosphate inhibits pyrophosphate hydrolysis. 32-Pyrophosphate (^32^PPi) hydrolysis in rat aortic rings cultured in MEM (without phosphate) supplemented (+Pi) or not (−Pi) with 1 mmol/L phosphate. (**A**) Representative ^32^PPi hydrolysis in the indicated time. (**B**) ^32^PPi hydrolysis in absence or presence of 1 mmoL/L phosphate after 20 min of incubation. Results are presented as mean ± SEM of 12 rings (in three independent experiments), and were compared using the Wilcoxon matched pairs test. ***P < 0.001.

For pyrophosphate hydrolysis experiment (Fig. 6), aortic rings were cultured *ex vivo* in Minimum Essential Medium Eagle (MEM Media, Gibco, Paisley, United Kingdom). To remove adventitia layer, rat aortas were digested for 10 min with collagenase, as previously described^28^. Then, medial layer of the aortic rings were incubated *ex vivo* in MEM media containing 5 µmol/L pyrophosphate and 32-pyrophosphate (^32^PPi) as a radiotracer. After the indicated time of incubation, ortophosphate was separated from pyrophosphate, as previously described^15^. Briefly, 20 μL of sample was mixed with 400 μL of ammonium molybdate (to bind the orthophosphate, 09913, Sigma-Aldrich) and 0.75 mol/L sulphuric acid (258105, Sigma-Aldrich). Samples were then extracted with 800 μL of isobutanol/petroleum ether (4:1) to separate the phosphomolybdate from the pyrophosphate (ref. 77379 and 360465 for petroleum ether and isopropanol, respectively; Sigma-Aldrich). Next, 400 μL of the organic phase containing phosphomolybdate was removed and subjected to radioactivity counting.

In experiments shown in Fig. 6, pyrophosphate hydrolysis in absence of phosphate assay was first performed. In this case, the aortic rings were incubated in MEM media without phosphate. Then, after washing five times in MEM media without phosphate, the same aortic rings were used for pyrophosphate hydrolysis assay in presence of 1 mmol/L phosphate (KH_2_PO_4_/K_2_HPO_4_ pH 7.4). Finally, the aortic rings were dried and weighed.

### Statistical analysis

Results are presented as mean ± standard error of the mean (SEM), and were compared by the Wilcoxon matched pairs test. Statistical significance was determined using GraphPad Prism 5 software.

## Electronic supplementary material

 Supplementary Information
