# Supplementary material for: Hydrolysis of Extracellular Pyrophosphate increases in post-hemodialysis plasma
Source: Sci Rep. 2018 Jul 23;8:11089. doi: 10.1038/s41598-018-29432-4 (PMC6056505; doi:10.1038/s41598-018-29432-4)
Supplement: Supplementary file 1 — Supplementary Information [file 41598_2018_29432_MOESM1_ESM.docx]

Hydrolysis of Extracellular Pyrophosphate increases in post-hemodialysis plasma

Daniel Azpiazu, Emilio González-Parra, Jesús Egido, Ricardo Villa-Bellosta

**Complete immunoblot (figure 5C)**

**
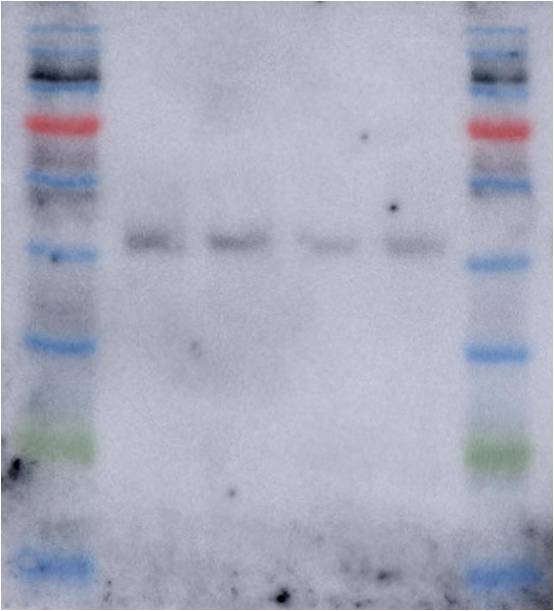
**
